# Entropy-Driven Inclusion of Natural Protoberberine Alkaloids in Sulfobutylether-β-Cyclodextrin

**DOI:** 10.3390/molecules27217514

**Published:** 2022-11-03

**Authors:** Zsombor Miskolczy, Mónika Megyesi, László Biczók

**Affiliations:** Institute of Materials and Environmental Chemistry, Research Centre for Natural Sciences, P.O. Box 286, 1519 Budapest, Hungary

**Keywords:** thermodynamics, host-guest binding, inclusion complex, self-assembly, macrocycle, fluorescence, calorimetry, drug delivery, alkaloid

## Abstract

The understanding of the relationship between molecular structure and the thermodynamics of host-guest binding is essential for the rational design of the applications of inclusion complexes. To obtain insight into the factors governing the driving force of complex formation in aqueous solutions, the encapsulation of five pharmaceutically important protoberberine alkaloids was studied in sulfobutylether-β-cyclodextrin having on average 6.4 degrees of substitution (SBE_6.4_βCD). Spectrophotometric, fluorescence spectroscopic, and isothermal calorimetric measurements showed 1:1 complexation in dilute solutions. From 1.92 × 10^4^ M^−1^, about an eight-fold decrease of the association constant was observed in the series of berberine ≈ coptisine >> palmatine > epiberberine > dehydrocorydaline. The embedment of these alkaloids in the SBE_6.4_βCD cavity was entropy-controlled with mildly negative enthalpy contributions. These findings suggest that the stabilization of the examined complexes arises primarily from the hydrophobic interaction between the constituents. The more than three orders of magnitude smaller association constants of protoberberine alkaloids with SBE_6.4_βCD than with cucurbit[7]uril, a host having similar cavity size, originates from the much smaller exothermicity of the confinement in the former macrocycle.

## 1. Introduction

Cyclodextrins are cyclic oligosaccharides widely used in the pharmaceutical, cosmetic, food, and textile industries [1,2,3,4]. Encapsulation in their hydrophobic cavity enhances the solubility, stability, and bioavailability of various compounds, and can reduce the unpleasant taste, toxicity, or side effects of drugs [3,5,6]. Among the natural cyclodextrins, the β-homologue (β-CD) composed of seven glucopyranose units is the most popular molecular container because of the good match of its interior with the size of many simple organic compounds. However, the poor (18.5 g dm^−3^) water-solubility of β-CD [7] represents a significant disadvantage in lots of applications. The attenuation of the intermolecular hydrogen bonding network among β-CD molecules in the crystal state by the substitution of the hydroxyl groups enormously improves the aqueous solubility [8]. The introduction of sulfobutylether groups is particularly beneficial because the hydrophilic sulfonate moiety not only augments solubility but also enables electrostatic attraction with cationic guests at all relevant pH values due to its anionic character. In addition, the butyl ether chain extends the hydrophobic surface of β-CD. The increase of the number of sulfobutylether substituents on the β-CD portals was found to enhance the binding affinity for the cationic LDS-798 dye [9] but does not lead to a uniform trend in the case of uncharged guests because the entropy increase compensates the less exothermic complexation [10]. The preferred degree of substitution is on average 6.5 sulfobutylether moiety per β-CD unit [11]. This sulfobutylether-β-cyclodextrin derivative is employed as an excipient in numerous marketed drug products and in formulations under development [11].

The encapsulation of biologically active plant alkaloids in sulfobutylated βCD has attracted particular attention. The combined use of this macrocycle and an ionic liquid facilitated the separation and quantification of four camptothecin-type anticancer alkaloids by capillary zone electrophoresis [12]. When a vinca alkaloid derivative, vinpocetine served as a guest, the inclusion efficiency was improved by the addition of polyvinylpyrrolidone or hydroxypropyl methylcellulose [13]. Sulfobutylation of β-CD brought about a 750-fold increase in the association constant for the complexation of mitragynine tetracyclic indole alkaloid [14]. The host-guest binding modified the photophysical properties and the antibacterial activity of sanguinarine [15]. Berberine exhibited high affinity to β-CD possessing 10 sulfobutylether arms. The strong 1:1 association induced ~190-fold fluorescence intensity enhancement, which was quenched by ternary complex formation with Cd^2+^ ion [16].

**Figure 1:**
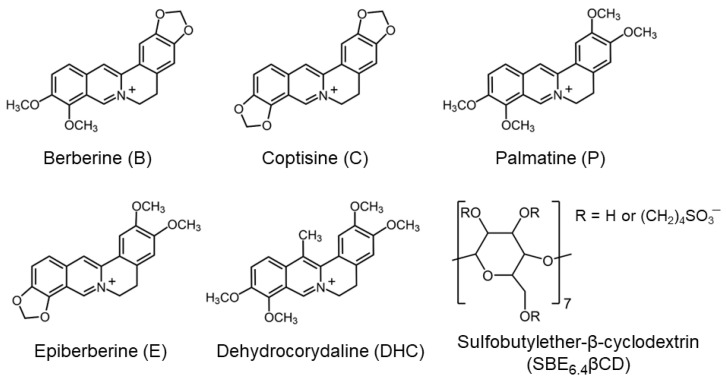
The structural formula of the guest and host compounds.

Despite the many applications of sulfobutylated βCD, few isothermal titration calorimetry studies have been performed to gain deeper insight into the factors controlling the strength of guest binding in its cavity [10,17,18,19,20,21,22,23]. The effect of systematic molecular structure variation on the thermodynamics of the inclusion of aromatic compounds in this macrocycle has not been examined. To fill this knowledge gap, we unravel how the minor alteration of the substitution pattern of the clinically important protoberberine alkaloids (Figure 1) influences the enthalpy and entropy changes upon inclusion complex formation. We have chosen to use the pharmaceutically relevant derivative that contains on average 6.4 sulfobutylether substituents per macrocycle (SBE_6.4_βCD). The results will be compared with those previously published for the entrapment in the rigid, nonpolar and extremely nonpolarizable core of cucurbit[7]uril (CB7), a host having similar cavity dimensions.

## 2. Results

### 2.1. Inclusion Complex Formation of Berberine with SBE_6.4_βCD

**Figure 2:**
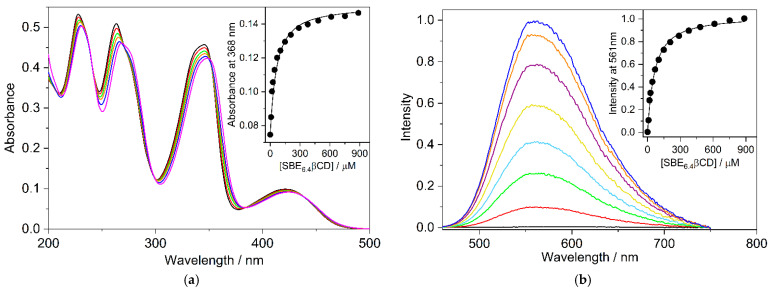
(**a**) Variation of the absorption spectrum of 19.3 μM B aqueous solution upon addition of 0 (black), 9.4 (red), 18 (green), 44 (orange), 102 (blue), and 886 μM (magenta) SBE_6.4_βCD at 10 mm optical path. Inset: Absorbance at 368 nm as a function of M SBE_6.4_βCD concentration (•); (**b**) Fluorescence spectra of 19.3 μM B aqueous solution in the presence of 0 (black), 9.4 (red), 18 (green), 44 (pale blue), 102 (ochre), 289 (purple), 886 (orange), and 1800 μM (blue) SBE_6.4_βCD. Excitation occurred at 382 nm. Inset: Fluorescence intensity at 561 nm as a function of SBE_6.4_βCD concentration (•). The experiments were performed at 298 K. The line in the insets stands for the fitted function.

The absorption and fluorescence spectra of B markedly change upon the gradual increase of SBE_6.4_βCD concentration (Figure 2). The absorption bands shift toward a longer wavelength and show slight hypochromicity in the presence of SBE_6.4_βCD. These spectral modifications are evidence of host-guest binding. The isosbestic points imply that only a single type of complex is produced in the employed concentration range. The absorbance alteration with the amount of SBE_6.4_βCD can be described well assuming 1:1 complexation and the line in the inset to Figure 2a represent the result of the nonlinear least squares analysis corresponding to K = (2.0 ± 0.1) × 10^4^ M^−1^ association constant. The Supplementary Materials provide the fitted functions [24].

The negligible fluorescence of B in water significantly grows, and a limiting value is approached when the SBE_6.4_βCD concentration is progressively raised (Figure 2b). The fluorescence quantum yield of B–SBE_6.4_βCD complex was found to be Φ_F_ = 0.11. The considerable fluorescence intensity enhancement is attributed to the partial immersion of B in the hydrophobic cavity of SBE_6.4_βCD, which diminishes the interaction with water and leads to a less polar microenvironment. The analysis of the fluorescence intensity variation provides K = (1.98 ± 0.10) × 10^4^ M^−1^ for the association constant of 1:1 embedment of B into SBE_6.4_βCD in good agreement with the value derived from spectrophotometric measurements. Fluorescence titration was also performed at lower (4.2 μM) B concentration where multiple alkaloid binding was less probable. The experimental data in Figure S1 in the Supplementary Materials show that the association constant (K = (1.92 ± 0.10) × 10^4^ M^−1^) remains unchanged within the limit of experimental errors in dilute B solution confirming 1:1 complexation stoichiometry. The K value obtained in the present study is considerably smaller than the quantity (2.67 × 10^5^ M^−1^) reported for B incorporation in SBE_10_βCD, a host possessing 10 sulfobutylether groups [16]. This is in accord with the predicted decline in the binding strength of a cationic guest with the degree of substitution on βCD [9].

**Figure 3:**
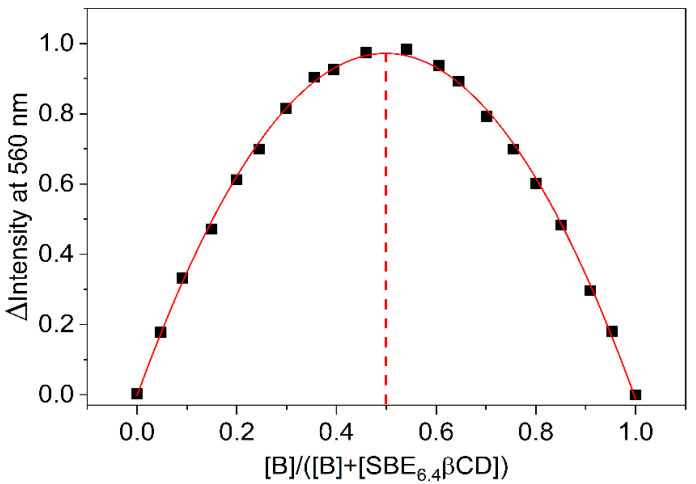
Job plot of the fluorescence intensity change at 560 nm as a function of the mole fraction of B (▪). The line displays the fitted second-order polynomial. The sum of SBE_6.4_βCD and B total concentrations is held constant (4.6 μM). Dash line shows 0.5 mole fraction.

Job’s method of continuous variations is widely used to establish the stoichiometry of self-assembly [25,26]. The concentration of the reactants was held constant (4.6 μM) and the relative host and guest amounts were systematically varied. The fluorescence intensity corrected by the B fluorescence was determined at 560 nm for each mixing ratio and the results are displayed as a function of SBE_6.4_βCD mole fraction in Figure 3. Analogous relationships were obtained at other monitoring wavelengths. The fit of the experimental data with a second-order polynomial provided a maximum at 0.50 mole fraction confirming 1:1 binding. The location of the maximum was invariant when the experiments were conducted at one order of magnitude larger (46 μM) constituent concentration (Figure S2 in the Supplementary Materials). The large fluorescence quantum yield of the complexed B (Φ_F_ = 0.11) allowed the accurate Job plot determination in dilute solutions. The correction by the fluorescence of the free B was always very small because of its feeble emission in water. Φ_F_ = 4.7 × 10^−4^ was found for the unbound B in D_2_O [27].

**Figure 4:**
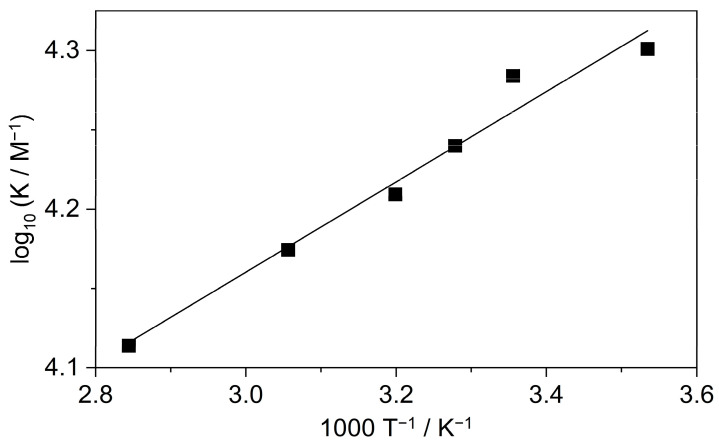
The van’t Hoff plot of the association constant of B confinement in SBE_6.4_βCD. The line displays the result of the nonlinear least-squares fit of the temperature dependence of K values by Equation (1).

To gain information on the thermodynamics of B inclusion in SBE_6.4_βCD, the association constant of the process was determined by the fluorescence method at various temperatures (T). The obtained binding isotherms are presented in Figures S1 and S3–S6 in the Supplementary Materials, while the logarithm of the association constants is plotted as a function of the reciprocal temperature in Figure 4.

The enthalpy (ΔH) and entropy changes (ΔS) of complexation were calculated from the K values based on the relationship:

(1) $$K=\left[\exp\left(\frac{\Delta S}{R}\right)\right]\left[\exp\left(-\frac{\Delta H}{\mathrm{RT}}\right)\right]$$

where R stands for the gas constant. The host-guest binding was found to be entropy-controlled with ΔS = 63 ± 4 J mol^−1^ K^−1^. The enthalpic contribution to the driving force was only ΔH = −5.4 ± 0.5 kJ mol^−1^.

**Figure 5:**
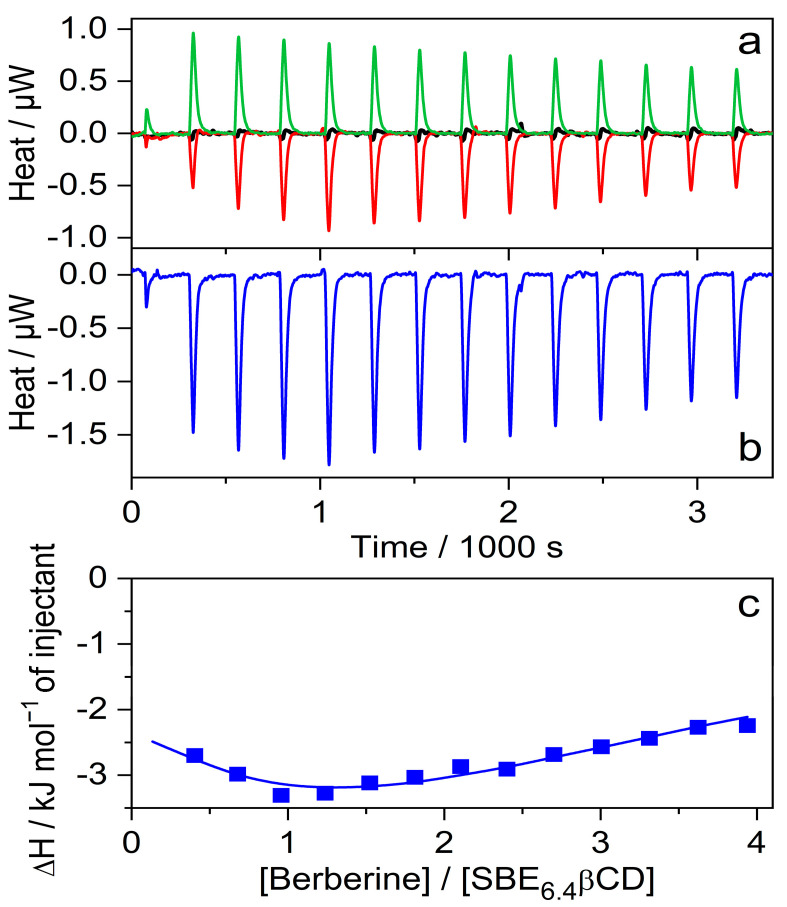
(**a**) ITC traces for the titration of 41 μM SBE_6.4_βCD solution with water (black line) and 790 μM B solution (red line). Absorbed heat upon the addition of 790 μM B solution into water (green line); 20 μL aliquots were injected into 1.433 mL volume. (**b**) Evolved heat after subtraction of the dilution heats of B and SBE_6.4_βCD. (**c**) The integral of the peaks displayed in panel (**b**) is divided by the mole of B as a function of [B]/[SBE_6.4_βCD] molar ratio. The line shows the best fit with a stepwise binding to two sites model.

Isothermal titration calorimetry (ITC) studies were performed to determine directly the ΔH of inclusion. Figure 5a shows the enthalpograms. The microcalorimeter cell of 1.433 mL volume was filled with 41 μM SBE_6.4_βCD solution and 20 μL aliquots of 790 μM B solution (red line) were added. As expected, a negligible signal appeared when the same amounts of water were injected (black line) instead of B solution because of the small extent of SBE_6.4_βCD dilution. The addition of 790 μM B solution to water generated weak endothermic signals (green line) which may be attributable to the dilution-induced dissociation of the small number of B dimers produced at such a high concentration. An analogous phenomenon has been found for quaternary benzo[c]phenanthridine alkaloids [28]. Figure 5b shows the calorimetric trace after the subtraction of the dilution heats. The integrals of the peaks represent the evolved heat stemming from the complexation after the sequential injections. Figure 5c displays the variation of the molar enthalpy change with increasing [B]/[SBE_6.4_βCD] molar ratio. The experimental data fit well to the model composed of the consecutive formation of 1:1 and 1:2 host-guest complexes with association constants and enthalpy changes of K_1_ = 1.8 × 10^4^ M^−1^, ∆H_1_ = −5.5 kJ mol^−1^ and K_2_ = 5.9 × 10^3^ M^−1^, ∆H_2_ = −27.1 kJ mol^−1^, respectively. The K_1_ and ∆H_1_ values agree with the corresponding quantities found by the fluorescence spectroscopic method (vide supra) at lower B concentrations where the binding of the second B plays an unimportant role. These results demonstrate that not only a 1:1 complex is created when the concentration of B is significantly larger than that of SBE_6.4_βCD.

**Figure 6:**
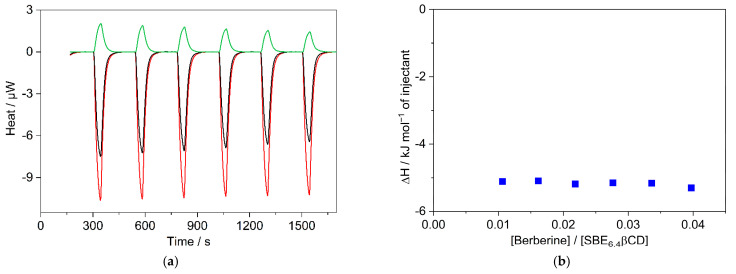
(**a**) ITC traces for the titration of 6.04 mM SBE_6.4_βCD solution with water (black line) and 1.12 mM B solution (red line). Absorbed heat upon the addition of 1.12 mM B solution into water (green line); 40 μL aliquots were injected into 1.433 mL volume. (**b**) The integral of the evolved heat after subtraction of the dilution heats of B and SBE_6.4_βCD as a function of [B]/[SBE_6.4_βCD] molar ratio.

To measure selectively the enthalpy change of the 1:1 confinement with great accuracy, SBE_6.4_βCD was always kept in substantial excess over B during the calorimetric experiment. SBE_6.4_βCD concentration of 6.04 mM was employed to ensure that 99% of B produced inclusion complex after each injection of 40 μL aliquots of 1.12 mM B solution. Figure 6a presents the enthalpograms for the raw experimental data for the complexation and the dilution of the constituents. After subtraction of the dilution heats and integration of the difference of the signals, the ∆H of 1:1 association is obtained at various [B]/[SBE_6.4_βCD] molar ratios (Figure 6b). The results do not vary within the limits of experimental errors because each added amount of B becomes practically fully complexed. Therefore, the mean of the ∆H values, −5.2 ± 0.2 kJ mol^−1^ gives the enthalpy change upon B encapsulation in SBE_6.4_βCD. Within the limit of experimental errors, the same ∆H is measured in more diluted solutions (Figure S7 in the Supplementary Materials) and the ITC results are in accordance with the corresponding data derived from the van’t Hoff plot of the association constants (Figure 4).

### 2.2. Effect of Alkaloid Substituent Variation on the Complexation with SBE_6.4_βCD

**Figure 7:**
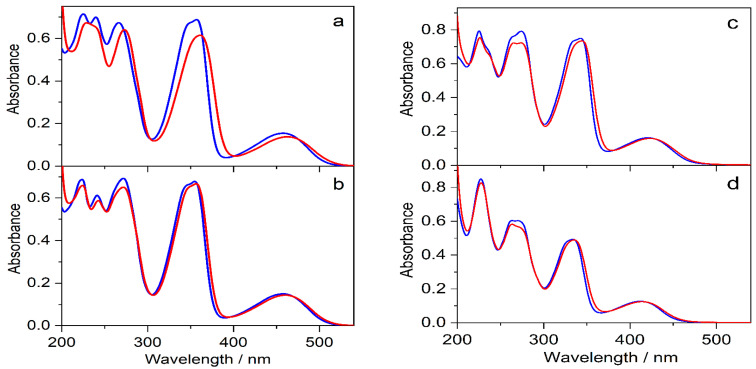
Absorption spectra of (**a**) 31 μM C, (**b**) 28 μM E, (**c**) 30 μM P, and (**d**) 22 μM DHC aqueous solutions (blue lines) and after the addition of 10 mM SBE_6.4_βCD (red lines) in quartz cuvette of 10 mm optical path.

**Figure 8:**
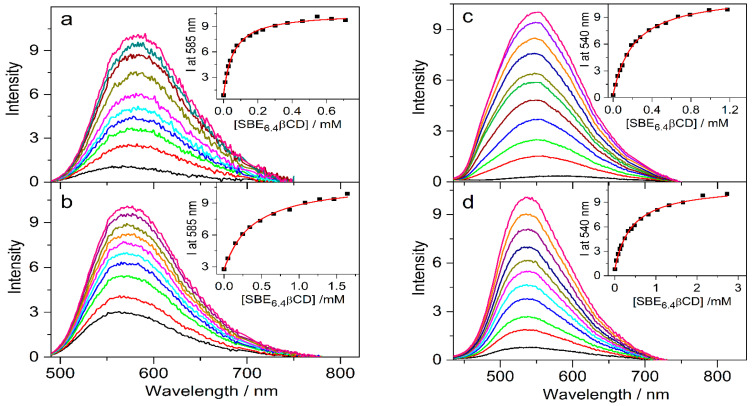
Fluorescence spectra of (**a**) 4.4 μM C, (**b**) 4.3 μM E, (**c**) 4.2 μM P, and (**d**) 4.6 μM DHC in water and in the presence of various amounts of SBE_6.4_βCD. Insets display the SBE_6.4_βCD concentration dependence on the fluorescence intensity (▪) and the lines present the fitted function.

The effects of 0.01 M SBE_6.4_βCD addition on the absorption spectra of protoberberine alkaloids are compared in Figure 7. In the case of C, the changes are similar to those observed for berberine. In contrast, the other three guest compounds show less pronounced spectral alterations upon confinement in SBE_6.4_βCD implying weaker binding. The association constants were determined by fluorescence titrations. A progressive increase in host concentration brought about a substantial fluorescence intensity rise (Figure 8) because the alkaloids sense a less polar microenvironment within the cavitand. The insets in Figure 8 demonstrate that the functions obtained by the nonlinear least-squares fit of the 1:1 binding model always match the experimental data well. The calculated association constants (K) are summarized in Table 1. B and C, the alkaloids possessing 2,3-dioxole ring but different substituents on their isoquinoline moiety, exhibit practically the same affinity to SBE_6.4_βCD. When two methoxy groups are introduced at the 2,3 positions of the alkaloids, the association constants become more than 4-fold smaller irrespective of the substitution pattern of the isoquinoline segment.

The enthalpy changes upon the 1:1 encapsulation of various alkaloids in SBE_6.4_βCD (∆H) were determined by ITC experiments as described for B (vide supra). The solution of the host compound served as a titrand, whose concentration was adjusted to ensure more than 98% complexation of the titrant alkaloid. To assure that only 1:1 confinement takes place, [alkaloid]/[ SBE_6.4_βCD] < 0.04 molar ratio was typically employed. The calorimetric results are presented in Figures S7–S11 in the Supplementary Materials and the mean ∆H values are listed in Table 1 together with the entropy changes (∆S) calculated as follows:

∆S = ∆H/T + R ln K(2)

where T = 298 K and R stands for the gas constant. The entropy term has the dominant contribution to the driving force of the 1:1 inclusion in SBE_6.4_βCD for all studied alkaloids. The larger binding affinity of B and C is attributed to their more exotherm complexation. For the sake of comparison, Table 1 also includes the corresponding quantities available in the literature for the embedment in CB7 [29,30,31].

## 3. Discussion

The association constant of B confinement in SBE_6.4_βCD (Table 1) is ~200-fold larger than the 87 M^−1^ value reported for the inclusion in the unsubstituted βCD at 298 K [32]. The difference is attributed to the fundamentally different thermodynamics of the two processes. Recalculation of the published temperature dependence of the association constants [32] provided ∆H = −33.4 kJ mol^−1^ and ∆S = −73.5 J mol^−1^ K^−1^ demonstrating that the exothermic embedment in βCD is accompanied by a highly unfavorable entropy change leading to enthalpy-driven complexation with a small driving force. The ∆H and ∆S quantities indicate tight binding of B in βCD, which results in a substantial enthalpy gain due to strong host-guest interaction and the release of energy-rich water from the host interior. The concomitant restriction of the movement of the constituents upon complexation causes a significant loss of entropy. In sharp contrast, we find a high entropy benefit upon B embedment in SBE_6.4_βCD suggesting that water molecules are expelled not only from the hydrophobic core of the macrocycle and from the surroundings of B but also from the hydration shells of the sulfobutylether substituents. The considerable enthalpy cost of desolvation is only slightly overcompensated by the enthalpy benefit of the host-guest interactions as shown by the small exothermicity of B–SBE_6.4_βCD production.

The data listed for various alkaloids in Table 1 show moderate variation with the molecular structure. Hence, the orientation of the examined alkaloids in SBE_6.4_βCD is not fundamentally different. The significant entropy growth plays the predominant role in the stabilization of all studied protoberberine alkaloid–SBE_6.4_βCD complexes. The alteration of the alkaloid substitution pattern brings about insignificant ∆S variation. The slightly lower ∆S for DHC is probably explained by the limitation of the degrees of freedom of the methyl group located at position 13 upon complexation. The other studied alkaloids do not have this substituent. The enthalpy release reaches only less than ~30% of the entropy term (−T∆S). The largest association constants appear for B and C due to the most favorable practically identical thermodynamic parameters of their insertion in SBE_6.4_βCD. These alkaloids contain a dioxole ring in positions 2,3 and differ only in the substituents of the isoquinoline heterocycle. The introduction of the bulkier pair of methoxy groups at positions 2,3 instead of the smaller dioxole group results in weaker affinity to SBE_6.4_βCD and smaller enthalpy diminution upon binding due probably to steric reasons. The alteration of the substituents on the isoquinoline part has a lesser impact on ∆H. The entropy-promoted formation of the alkaloid–SBE_6.4_βCD complexes is contrary to the findings that the vast majority of CD complexes are produced in enthalpy-driven processes [33]. The different behavior is attributed to the enhanced hydrophobic interaction and the possibility of ion pairing. The liberation of water molecules from the apolar surfaces of the butylene chains and the core of SBE_6.4_βCD as well as from the vicinity of the alkaloid ring system give rise to a substantial entropy increase. The ion pairing is generally also accompanied by the release of water molecules from the hydrate shell of the interacting oppositely charged constituents leading to favorable entropic terms [34]. For example, an average of −T∆S = −8 ± 2 kJ mol^−1^ was found for the ion pairing of alkali salts [35].

It is worth comparing the data for the alkaloid inclusion in SBE_6.4_βCD with the corresponding quantities published for the complexation with CB7 (Table 1). The association constants of CB7 are more than three orders of magnitude larger than those of SBE_6.4_βCD because of the much more exotherm incorporation in the former cavitand. The energetic frustration of water molecules is more substantial in the CB7 cavity than in the SBE_6.4_βCD interior because of three main reasons. (i) The polarizability inside CB7 (P = 0.12) is extremely low, and falls in between that of perfluorohexane (P = 0.159) and the gas phase (P = 0.000) [36]. In contrast, a P = 0.20 value is reported for the core of βCD, which barely differs from the polarizability of water (P = 0.206) [37]. (ii) The hydrogen bonding of the entrapped water with the glycosidic oxygens and hydroxyl groups of the SBE_6.4_βCD macrocycle allows smaller distortion of the water structure from its optimal form in this host than in CB7 [38]. Therefore, the release of water from CB7 results in a larger enthalpy benefit. (iii) In addition, the βCD macrocycle is filled with less (on average 6.5) water [39] than CB7, which encompasses on average 7.9 water [40]. The larger number and the higher energy of the internal water molecules in CB7 result in more significant enthalpy gain upon their expulsion into the bulk by alkaloid guests than in the case of SBE_6.4_βCD. The larger fluorescence quantum yield of B in CB7 (Φ_F_ = 0.26) [41] than in SBE_6.4_βCD (Φ_F_ = 0.11 vide supra) probably indicates the more substantial desolvation upon inclusion in the former host. The tight binding in the rigid CB7 more efficiently restricts the degrees of freedom of the components than the association with the more flexible SBE_6.4_βCD. The more substantial entropy loss caused thereby for the complexation with CB7 better counterbalances the entropy gain originating from the release of the conformationally restricted water molecules from the host and guest hydrate shells. Consequently, alkaloid inclusion in CB7 is accompanied by a smaller entropy advantage than the confinement to SBE_6.4_βCD. To reach larger K and more negative ∆H, the location of the less voluminous dioxole substituent of the protoberberine alkaloids is very important. For the association with SBE_6.4_βCD, positions 2,3 is the preferable dioxole site as shown by the strongest binding of B and C (Table 1). In contrast, the alkaloids (C and E) possessing a dioxole ring at the position 9,10 are entrapped with the largest affinity and exothermicity in CB7.

## 4. Materials and Methods

Alkaloids were obtained as chloride salts. B and P (Sigma-Aldrich, Budapest, Hungary) were chromatographed on a silica gel (Merck, Budapest, Hungary) column eluting with ethanol (Sigma-Aldrich, Budapest, Hungary). C (CFM Oskar Tropitzsch GmbH, Marktredwitz, Germany), E (ChemFaces Biochemical Co., Wuhan, China), and DHC (BOC Sciences, Shirley, NY, USA) were used as received. SBE_6.4_βCD (Cyclolab, Budapest, Hungary) was delivered as a sodium salt. Capillary electrophoresis measurements demonstrated that the average degree of substitution with sulfobutylether groups was 6.40, while Karl-Fischer titrations showed a water content of 6.1%. Water was freshly distilled twice from dilute KMnO_4_ solution. Experiments were carried out at 298 K unless otherwise stated.

The UV-visible absorption spectra were acquired with a Cary60 (Agilent Technologies, Santa Clara, CA, USA) spectrophotometer using quartz cuvettes of a 10 mm optical path. Corrected fluorescence spectra were recorded on a Fluoromax-4 (Jobin-Yvon, Longjumeau, France) photon counting spectrofluorometer. The light of 330 nm wavelength served for the excitation of DHC solutions. Samples of B and P were excited at 345 nm, while the excitation wavelength was set to 350 nm for C and EB. Alkaloid aqueous solutions of ~20 or ~4 μM were mixed in various ratios with the stock solutions of 10 mM SBE_6.4_βCD containing the same amount of the corresponding alkaloid in water in the case of spectrophotometric or fluorescence spectroscopic titrations, respectively. The temperature was controlled with a Julabo (Seelbach, Germany) F25-ED thermostat. Job plots were created from fluorescence titration data following the previously published simplified experimental route [25]. Either 4.6 μM B or 4.6 μM SBE_10_βCD solution was titrated with equimolar reactant solution and the fluorescence intensity change was monitored at 560 nm. The intensity of B fluorescence was subtracted at each concentration and the results were plotted as a function of the alkaloid mole fraction. The fluorescence quantum yield of B–SBE_6.4_βCD complex was measured relative to that of quinine sulfate in 0.5 M H_2_SO_4_ solution, for which a reference yield of 0.546 was taken [42].

Isothermal titration calorimetry (ITC) studies were carried out with a MicroCal VP-ITC (GE Healthcare, Chicago, IL, USA) microcalorimeter at 298 K (cell volume 1.433 mL). In the typical experiments, alkaloid aqueous solution (0.7–1.5 mM) was injected stepwise (40 μL each, duration 40 s) from the computer-controlled microsyringe into 3–25 mM SBE_6.4_βCD in water with intervals of 240 s between the individual additions. To ensure proper mixing after each injection, a stirring speed of 307 rpm was applied. The first injection was not included in the data analysis. The dilution heats, measured by adding water into SBE_6.4_βCD or by injecting alkaloid solution into water, were subtracted from the integrated heat evolved per injection in the titration of SBE_6.4_βCD under the same conditions. The results obtained at [alkaloid]/[SBE_6.4_βCD] < 0.04 molar ratios were averaged.

## 5. Conclusions

In contrast with the enthalpy-promoted formation of the majority of cyclodextrin complexes [33], the entropic contribution to the Gibbs free energy diminution dominates for protoberberine encapsulation in SBE_6.4_βCD. The substantial entropy gain implies that the hydrophobic interactions prevail in the stabilization of the produced complexes. The improved binding ability upon linking sulfobutylether groups to βCD macrocycle originates from the substantial ∆S augmentation for host-guest binding. Due to its much larger solubility and complexation efficiency, SBE_6.4_βCD can be a better drug solubilizer than its parent compound, βCD [43]. The entropy increase may also play an important role in the formation of cyclodextrin-based polyrotaxanes possessing hydrophilic guest units [44,45].

## Figures and Tables

**Table 1:** Binding constants and thermodynamic parameters for the 1:1 encapsulation of protoberberine alkaloids in SBE_6.4_βCD and cucurbit[7]uril macrocycles at 298 K in water.

Host Macrocycle		Berberine	Coptisine	Palmatine	Epiberberine	Dehydrocorydaline
SBE_6.4_βCD	K/10^3^ M^−1^	19.2 ± 1.0	19.1 ± 1.3	4.7 ± 0.2	3.0 ± 0.3	2.5 ± 0.2
	∆H/kJ mol^−1^	−5.2 ± 0.2	−5.7 ± 0.3	−2.6 ± 0.4	−1.9 ± 0.3	−3.2 ± 0.4
	−T∆S/kJ mol^−1^	−19.2 ± 1.8	−18.7 ± 1.9	−18.3 ± 1.6	−17.9 ± 1.8	−16.2 ± 1.7
	∆S/J mol^−1^ K^−1^	64 ± 6	63 ± 6	62 ± 5	60 ± 6	54 ± 6
Cucurbit[7]uril ^a,b^	K/10^7^ M^−1^	2.4 ± 0.3 ^a^	50 ± 15 ^a^	2.6 ± 0.3 ^a^	62 ± 15 ^a^	0.21 ± 0.03 ^b^
	∆H/kJ mol^−1^	−38 ± 2 ^a^	−45 ± 2 ^a^	−37 ± 2 ^a^	−45 ± 2 ^a^	−34 ± 1 ^b^
	−T∆S/kJ mol^−1^	−4.5 ± 0.1 ^c^	−6.9 ± 2.1 ^c^	−2.7 ± 1.2 ^c^	−7.2 ± 2.1 ^c^	−1.8 ± 0.6 ^c^
	∆S/J mol^−1^ K^−1^	15 ± 4 ^a^	23 ± 7 ^a^	9 ± 4 ^a^	24 ± 7 ^a^	6 ± 2 ^b^

^a^ References [29,30], ^b^ Reference [31], ^c^ Calculated from the published ∆S values.

## Data Availability

Data supporting reported results can be found in the article and the Supplementary Materials.

## Supplementary Materials

The following supporting information can be downloaded at: https://www.mdpi.com/article/10.3390/molecules27217514/s1, Fitted functions, Figure S1: Fluorescence intensity change at 564 nm as a function of SBE_6.4_βCD concentration in 4.2 μM berberine aqueous solution at 298.0 K; Figure S2: Job plot of the fluorescence intensity change at 560 nm as a function of the mole fraction of B; Figures S3–S6: Fluorescence intensity growth upon the increase of SBE_6.4_βCD concentration in 4.2 μM berberine aqueous solution at 282.9, 312.6, 327.2, and 351.6 K; 1996243; Figure S7: (a) ITC traces for the titration of 3.00 mM SBE_6.4_βCD solution with water (black line) and 0.79 mM B solution (red line). Absorbed heat upon the addition of 0.79 mM B solution into water (green line); 40 βL aliquots were injected into 1.433 mL volume. (b) The integral of the evolved heat after subtraction of the dilution heats of B and SBE_6.4_βCD as a function of [B]/[SBE_6.4_βCD] molar ratio; Figure S8: (a) ITC traces for the titration of 6.00 mM SBE_6.4_βCD solution with water (black line) and 0.82 mM C solution (red line). Absorbed heat upon the addition of 0.82 mM C solution into water (green line); 40 μL aliquots were injected into 1.433 mL volume. (b) The integral of the evolved heat after subtraction of the dilution heats of C and SBE_6.4_βCD as a function of [C]/[SBE_6.4_βCD] molar ratio; Figure S9: (a) ITC traces for the titration of 16.2 mM SBE_6.4_βCD solution with water (black line) and 0.84 mM P solution (red line). Absorbed heat upon the addition of 0.84 mM P solution into water (green line); 40 μL aliquots were injected into 1.433 mL volume. (b) The integral of the evolved heat after subtraction of the dilution heats of P and SBE_6.4_βCD as a function of [P]/[SBE_6.4_βCD] molar ratio; Figure S10: (a) ITC traces for the titration of 20.1 mM SBE_6.4_βCD solution with water (black line) and 1.07 mM E solution (red line). Absorbed heat upon the addition of 1.07 mM E solution into water (green line); 40 μL aliquots were injected into 1.433 mL volume. (b) The integral of the evolved heat after subtraction of the dilution heats of E and SBE_6.4_βCD as a function of [E]/[SBE_6.4_βCD] molar ratio; Figure S11: (a) ITC traces for the titration of 24.8 mM SBE_6.4_βCD solution with water (black line) and 1.10 mM DHC solution (red line). Absorbed heat upon the addition of 1.10 mM DHC solution into water (green line); 40 μL aliquots were injected into 1.433 mL volume. (b) The integral of the evolved heat after subtraction of the dilution heats of DHC and SBE_6.4_βCD as a function of [DHC]/[SBE_6.4_βCD] molar ratio [24].
