# Supplementary material for: Entropy-Driven Inclusion of Natural Protoberberine Alkaloids in Sulfobutylether-β-Cyclodextrin
Source: Molecules. 2022 Nov 3;27(21):7514. doi: 10.3390/molecules27217514 (PMC9657192; doi:10.3390/molecules27217514)
Supplement: Supplementary file 1 [file molecules-27-07514-s001.zip › molecules-2001862-supplementary.pdf]

Supplementary Materials

Entropy-driven inclusion of natural protoberberine alkaloids in sulfobutylether-β-cyclodextrin

Zsombor Miskolczy, Mónika Megyesi, László Biczók*

Institute of Materials and Environmental Chemistry, Research Centre for Natural Sciences, P.O. Box 286, 1519 Budapest, Hungary

*Correspondance: biczok.laszlo@ttk.hu (László Biczók)

Fitted functions

The following function describes the relationship between the absorbance at a particular wavelength (A) and the total concentration of the host compound ($[SBE_6\beta CD]_0$) for 1:1 complex formation:[1]

$$A = A_0 + \frac{A_\infty - A_0}{2}\left\{1 + \frac{[SBE_6\beta CD]_0}{[B]_0} + \frac{1}{K[B]_0} - \left[\left(1 + \frac{[SBE_6\beta CD]_0}{[B]_0} + \frac{1}{K[B]_0}\right)^2 - 4\frac{[SBE_6\beta CD]_0}{[B]_0}\right]^{\frac{1}{2}}\right\} \qquad (S1)$$

where K represents the association constant, A_∞ and A_0 denote the absorbance of the fully complexed and free berberine, whose initial concentration is $[B]_0$.

In the case of 1:1 binding, the fluorescence intensity at a particular wavelength varies with the total concentration of the host compound ($[SBE_6\beta CD]_0$) as follows:[1]

$$I = I_0 + \frac{I_\infty - I_0}{2}\left\{1 + \frac{[SBE_6\beta CD]_0}{[B]_0} + \frac{1}{K[B]_0} - \left[\left(1 + \frac{[SBE_6\beta CD]_0}{[B]_0} + \frac{1}{K[B]_0}\right)^2 - 4\frac{[SBE_6\beta CD]_0}{[B]_0}\right]^{\frac{1}{2}}\right\} \qquad (S2)$$

where K represents the association constant, $[B]_0$ stands for the initial berberine concentration, I_∞ and I_0 denote the fluorescence intensity of the fully complexed and free berberine.

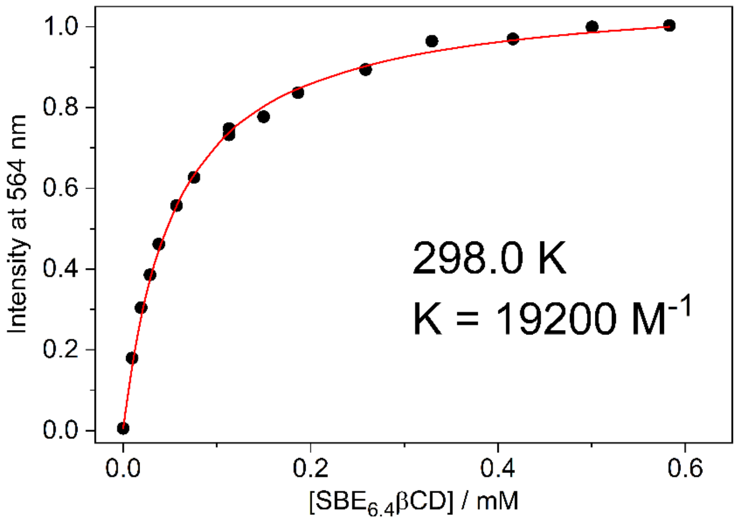

Figure S1. Fluorescence intensity change at 550 nm as a function of $SBE_{6.4}\beta CD$ concentration in 4.2 μM berberine aqueous solution at 298.0 K. Excitation wavelength was 345 nm. The line represents the fitted function corresponding to K = 19200 M^{-1} association constant for 1:1 complexation.

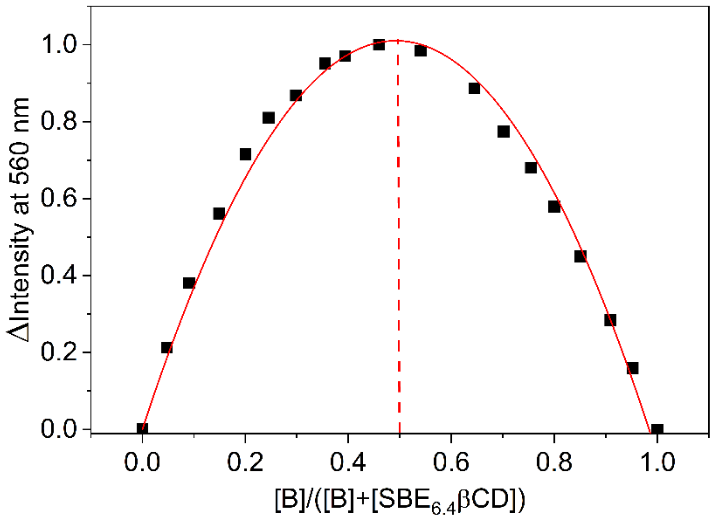

Figure S2. Job plot of the fluorescence intensity change at 560 nm as a function of the mole fraction of B. The sum of $SBE_{6.4}\beta CD$ and B total concentrations is held constant (46 μM). To avoid inner filter effect, excitation was performed at 380 nm where the absorbance is only 0.117 at 1 cm optical path.

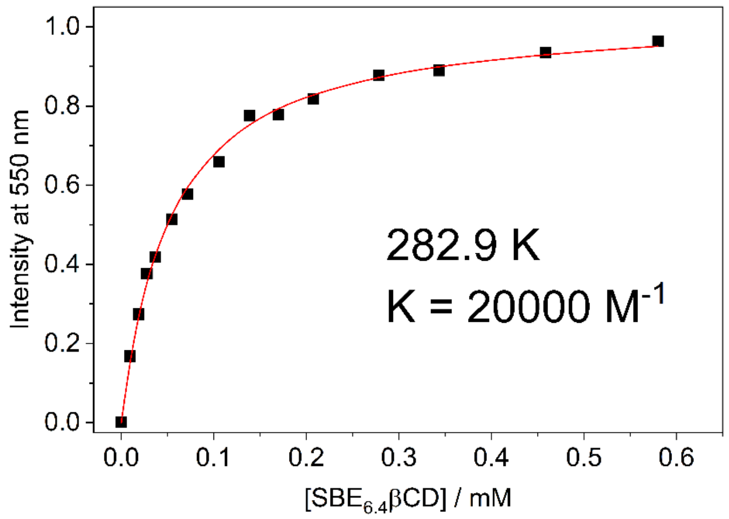

Figure S3. Fluorescence intensity growth upon increase of $SBE_6\beta CD$ concentration in 4.2 μM berberine aqueous solution at 282.9 K. Excitation wavelength was 345 nm. The line represents the fitted function corresponding to K = 20000 M^{-1} association constant for 1:1 complexation.

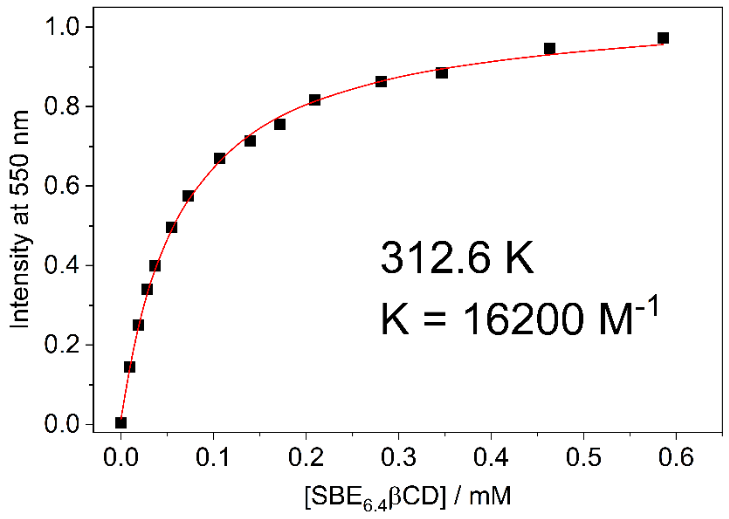

Figure S4. Fluorescence intensity growth upon increase of $SBE_6\beta CD$ concentration in 4.2 μM berberine aqueous solution at 312.6 K. Excitation wavelength was 345 nm. The line represents the fitted function corresponding to K = 16200 M^{-1} association constant for 1:1 complexation.

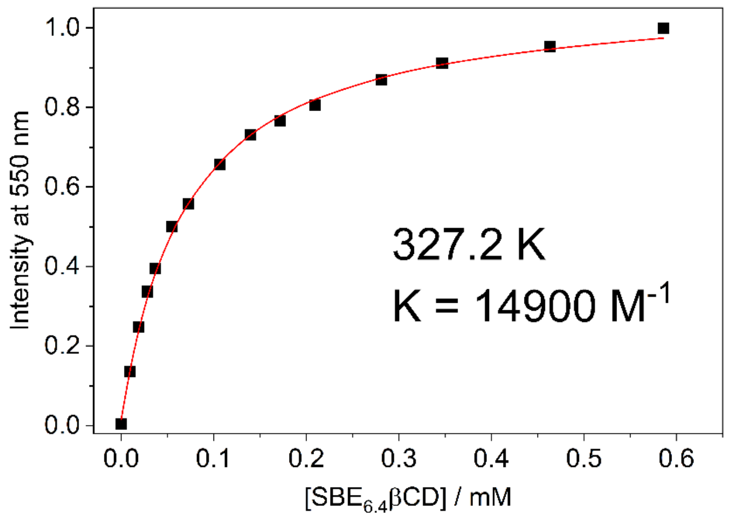

Figure S5. Fluorescence intensity growth upon increase of $SBE_6\beta CD$ concentration in 4.2 μM berberine aqueous solution at 327.2 K. Excitation wavelength was 345 nm. The line represents the fitted function corresponding to K = 14900 M^{-1} association constant for 1:1 complexation.

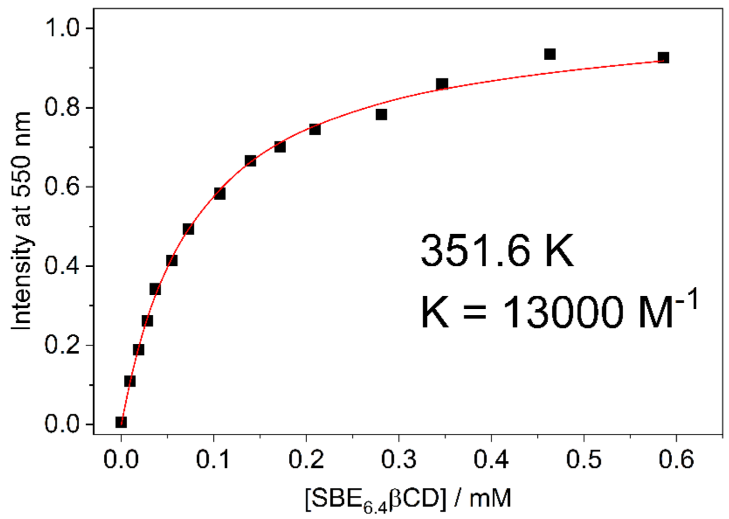

Figure S6. Fluorescence intensity growth upon increase of $SBE_6\beta CD$ concentration in 4.2 μM berberine aqueous solution at 351.6 K. Excitation wavelength was 345 nm. The line represents the fitted function corresponding to K = 13000 M^{-1} association constant for 1:1 complexation.

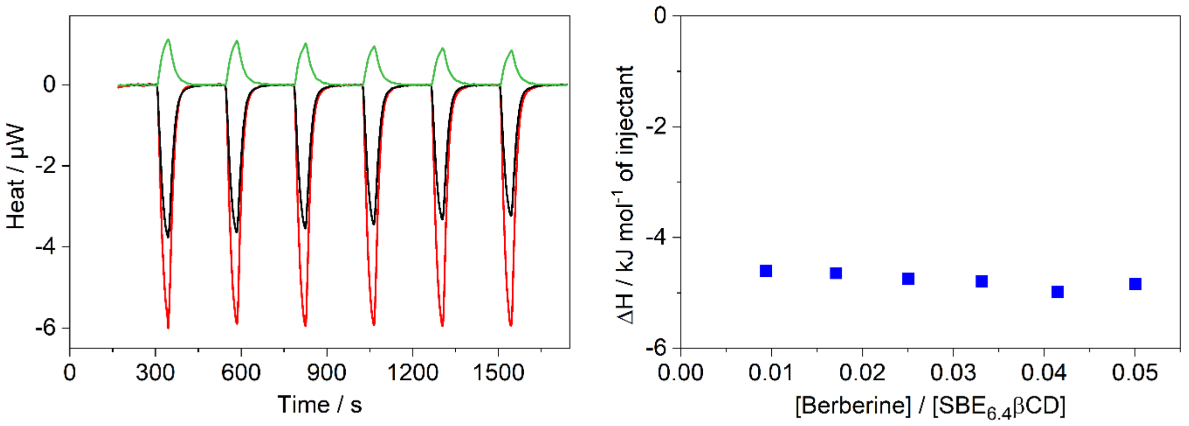

(a) **(b)**

Figure S7. (a) ITC traces for the titration of 3.00 mM $SBE_{6.4}\beta CD$ solution with water (black line) and 0.79 mM B solution (red line). Absorbed heat upon the addition of 0.79 mM B solution into water (green line); 40 µL aliquots were injected into 1.433 mL volume. (b) The integral of the evolved heat after subtraction of the dilution heats of B and $SBE_{6.4}\beta CD$ as a function of [B]/[$SBE_{6.4}\beta CD$] molar ratio.

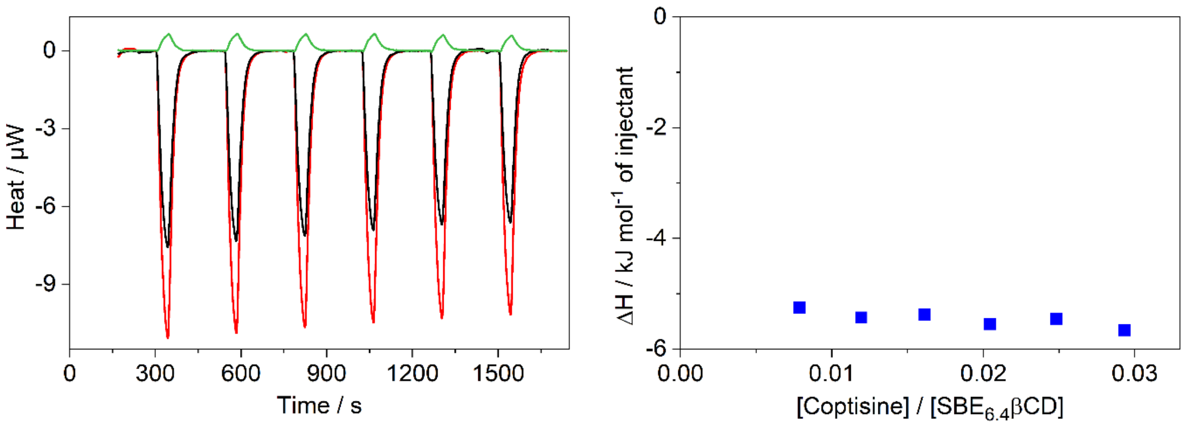

(a) **(b)**

Figure S8. (a) ITC traces for the titration of 6.00 mM $SBE_{6.4}\beta CD$ solution with water (black line) and 0.82 mM C solution (red line). Absorbed heat upon the addition of 0.82 mM C solution into water (green line); 40 µL aliquots were injected into 1.433 mL volume. (b) The integral of the evolved heat after subtraction of the dilution heats of C and $SBE_{6.4}\beta CD$ as a function of [C]/[$SBE_{6.4}\beta CD$] molar ratio.

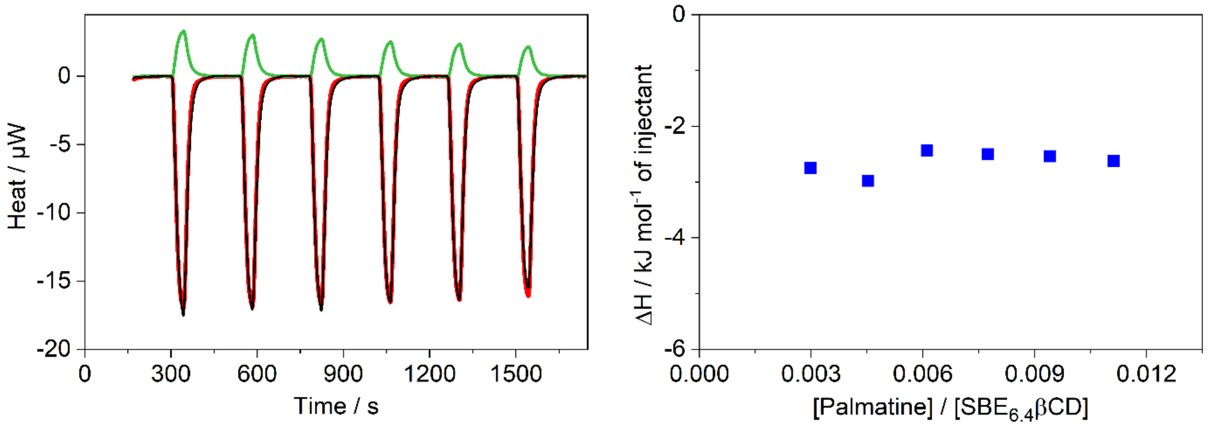

(a) **(b)**

Figure S9. (a) ITC traces for the titration of 16.2 mM SBE$_{6.4}$βCD solution with water (black line) and 0.84 mM P solution (red line). Absorbed heat upon the addition of 0.84 mM P solution into water (green line); 40 μL aliquots were injected into 1.433 mL volume. (b) The integral of the evolved heat after subtraction of the dilution heats of P and SBE$_{6.4}$βCD as a function of [P]/[SBE$_{6.4}$βCD] molar ratio.

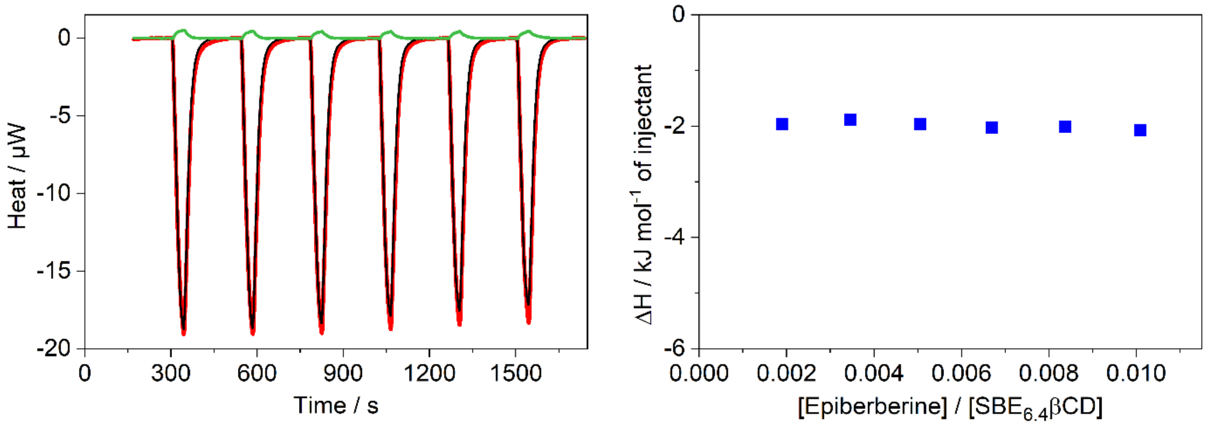

(a) **(b)**

Figure S10. (a) ITC traces for the titration of 20.1 mM SBE$_{6.4}$βCD solution with water (black line) and 1.07 mM E solution (red line). Absorbed heat upon the addition of 1.07 mM E solution into water (green line); 40 μL aliquots were injected into 1.433 mL volume. (b) The integral of the evolved heat after subtraction of the dilution heats of E and SBE$_{6.4}$βCD as a function of [E]/[SBE$_{6.4}$βCD] molar ratio.

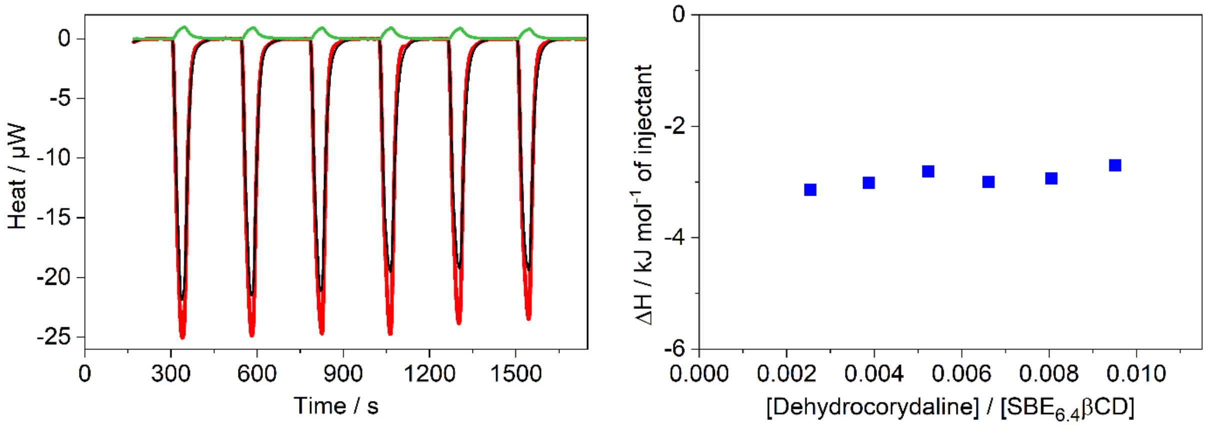

(a) **(b)**

Figure S11. (a) ITC traces for the titration of 24.8 mM $SBE_{6.4}\beta CD$ solution with water (black line) and 1.10 mM DHC solution (red line). Absorbed heat upon the addition of 1.10 mM DHC solution into water (green line); 40 μL aliquots were injected into 1.433 mL volume. (b) The integral of the evolved heat after subtraction of the dilution heats of DHC and $SBE_{6.4}\beta CD$ as a function of [DHC]/[$SBE_{6.4}\beta CD$] molar ratio.

Reference

1. Valeur, B. *Molecular Fluorescence, Principles and Applications*; Wiley-VCH: Weinheim, Germany, 2002; p. 343.
